# Five Years’ Experience in the New Cure of Consumption

**Published:** 1891-04

**Authors:** 


### BOOK NOTICES.

Five Years' Experience in the New Cure of Consumption by its own virus presumably on a line with the method of Koch. Illustrated by fifty-four cases. By J. Compton Burnett, M. D. London: The Homœopathic Publishing Co., 12 Warwick Lane, Paternoster Row, E. C. 1890.

The author of this little book is well known for his many clever monographs that arouse the interest of the profession from time to time.

The present volume is a record of cases treated with Tuberculinum, followed by the most favorable results. It is, therefore, a work of absorbing interest in these days when the attention of the whole medical world is centred upon Dr. Koch, and that of the homœopatic school in particular aroused by the claims of Dr. Samuel Swan to the origination of an improved method of applying the homœopathic principle.

Perhaps the best way of showing the position of the author would be by quoting from the preface the following words: "Wherever the cure of disease is concerned the practitioners of scientific Homœopathy have ever been in the van; and it is, therefore, not surprising that they should have been before all others in using the virus of consumption wherewith to cure consumption itself. But, a number of years ago, the leaders of the dominant sect of the medical profession raised a hue and cry against those of the homœopaths who were so unspeakable as to use the virus of consumption against the disease itself; and for fear of an unbearable amount of opposition and ignorant prejudice the practice was discountenanced and almost discontinued, a few only publishing here and there a striking case of the cure of consumption by the virus of the process itself.

"I am one of those on whom the opposition and ridicule have acted as an incentive for further observations and research, and for the past five years I have regularly used the bacillic virus as a part of my daily practice, and that in the aggregate with great satisfaction. Thus it is that the material that makes up this small treatise has been slowly accumulating." W. M. J.

